# Heterogeneity of presynaptic proteins: do not forget isoforms

**DOI:** 10.3389/fncel.2013.00008

**Published:** 2013-02-04

**Authors:** Luca Bragina, Giorgia Fattorini, Silvia Giovedì, Federica Bosco, Fabio Benfenati, Fiorenzo Conti

**Affiliations:** ^1^Department of Experimental and Clinical Medicine, Section of Neuroscience and Cell Biology, Università Politecnica delle MarcheAncona, Italy; ^2^Center for Neurobiology of Aging, Istituto Nazionale di Ricovero e Cura per AnzianiAncona, Italy; ^3^Department of Experimental Medicine, Università di GenovaGenova, Italy; ^4^Department of Neuroscience and Neurotechnologies, The Italian Institute of TechnologyGenova, Italy; ^5^Fondazione di Medicina Molecolare, Università Politecnica delle MarcheAncona, Italy

**Keywords:** GABA synapse, glutamate synapse, heterogeneity, SVs, isoforms

## Abstract

Analysis of presynaptic protein expression in glutamatergic and GABAergic central synapses performed in several laboratories and with different techniques is unveiling a complex *scenario*, largely because each presynaptic protein exists in several isoforms. The interpretation of these findings is generally based on the notion that each synapse and each synaptic vesicle contains one of the isoforms of each family of presynaptic proteins. We verified whether this interpretation is tenable by performing triple labeling and immunoisolation studies with the aim of detecting two isoforms of a given presynaptic protein in glutamatergic or GABAergic axon terminals and/or synaptic vesicles (SVs). Here, we show that: (1) the possibility that not all families of presynaptic proteins are expressed in all terminals must be taken into serious account; (2) the expression of a given protein isoform in a terminal does not exclude the expression of other isoforms of the same protein in the same terminal and in the same vesicle. These conclusions open new and interesting problems; their experimental analysis might improve our understanding of the physiology and pathophysiology of central synapses.

## Introduction

Neurotransmitter release is a fundamental process in synaptic communication, and heterogeneous expression of presynaptic proteins appears to contribute to functional differences, e.g., release probability, strength, and plasticity (Staple et al., 2000). Analysis of differential protein expression in central synapses has thus become an important research line in contemporary neuroscience (e.g., Sugino et al., 2006; O'Rourke et al., 2012), one of extraordinary difficulty given the elevated number of presynaptic proteins related to transmitter release and the existence of several isoforms of most of them. To date, most studies have focused on differential expression of these proteins in the predominant types of CNS synapses, i.e., glutamatergic and GABAergic (Conti and Weinberg, 1999; Cherubini and Conti, 2001).

In previous studies, we reported the heterogeneous expression of couples of isoforms (synapsin [SYN] I and II; synaptophysin [SYP] I and II; synaptosomal-associated protein [SNAP]-25 and SNAP-23; synaptogyrin [SGYR] 1 and 3; vesicle-associated membrane protein [VAMP] 1 and 2; syntaxin [STX] 1A and 1B, synaptotagmin [SYT] 1 and 2; synaptic vesicle protein [SV2] A and B, Rab3a and c) in vesicular glutamate transporter (VGLUT) 1−, VGLUT2− and vesicular GABA transporter (VGAT)-positive (+) axon terminals in rat cerebral cortex, and showed that VGLUT1+, VGLUT2+, and VGAT+ cortical axon terminals exhibit distinct expression profiles of presynaptic proteins (Bragina et al., 2007, 2010, 2012).

Whereas these observations provide information on the expression of each isoform in glutamatergic and GABAergic terminals, they leave the question of the relative expression of the two members of a couple in a given terminal unanswered. The case is well exemplified by the distribution of STX1A and 1B in VGLUT1+ terminals: ~60% of VGLUT1+ terminals express STX1A, while ~40% express STX1B (Bragina et al., 2010). Based on the classical notion that each synapse (and each vesicle) contains at least one isoform of each family of presynaptic proteins (Jahn and Südhof, 1994), the most likely interpretation for this observation is that each VGLUT1 terminal expresses either STX1A or STX1B. To verify whether this interpretation is tenable, we performed triple labeling studies in order to detect two isoforms (the most expressed and functionally meaningful ones) of a given presynaptic protein in glutamatergic and/or GABAergic axon terminals.

## Presynaptic proteins isoforms in glutamatergic and GABAergic terminals

**Figure 1:**
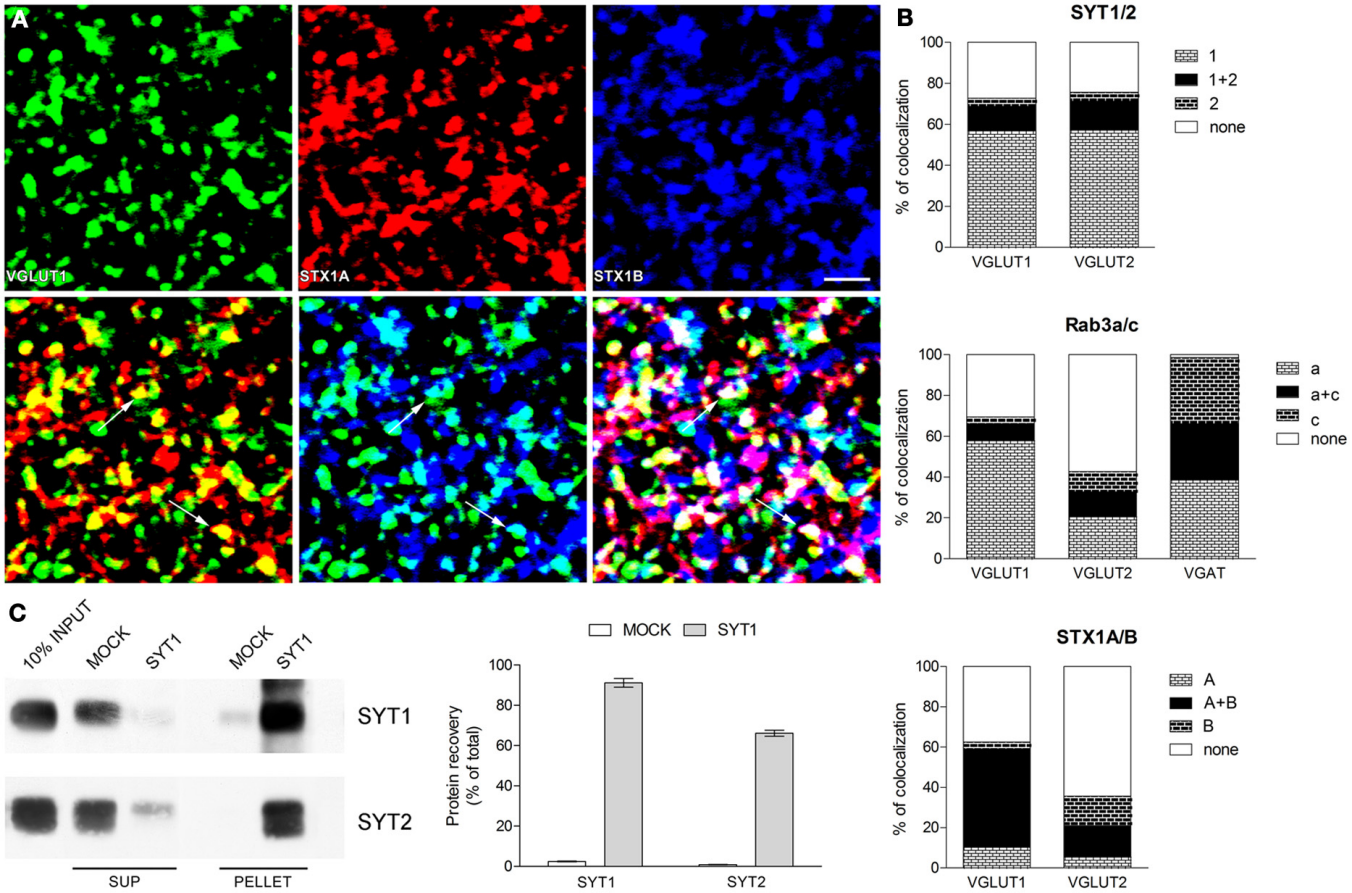
**Expression of presynaptic proteins isoforms in cerebral cortex (A) Triple labeling confocal microscopy studies.** Example of triple labeling studies: green codes for VGLUT1, red for STX1A and blue for STX1B. Rat brains (2–4 for each pairs of isoforms) were perfused and post-fixed for 2 h with 4% PFA (Bragina et al., 2007); vibratome sections (2–4 for each series) were processed with the same antibodies (to VGLUT1, VGLUT2, VGAT, SYT1 and 2, Rab3a and c, STX1A and B) and using the same conditions and methods described in previous studies (Bragina et al., 2007, 2010, 2012). Images were from parietal cortex and were acquired from randomly selected subfield (20–30 for each section) in layers II–VI. Each channel was examined separately to identify and manually count immunopositive puncta (first line); the two channels (green and red or green and blue) and the three channels were than merged and the number of co-localizing puncta was counted manually (second line). Following merging, puncta were considered double and triple-labeled when the overlap was complete or it occupied most of the area of the puncta and they were morphologically similar (arrows in second line). Bar 2 μm. **(B)** Co-localization of SYT, Rab3, and STX1 isoforms in VGLUT1+, VGLUT2+, and VGAT+ axon terminals in cerebral cortex. Graphs show isoforms colocalization in three population of axon terminals identified by the specific vesicular transporter they express. **(C)** Immunoprecipitation studies of SVs. SYT1 SVs were immunoisolated from the LS1 fraction of rat cerebral cortex using Eupergit C1Z beads coupled with SYT1 antibody or secondary antibody alone as negative control (MOCK). After immunoprecipitation pellet and supernatant (SUP) were subjected to immunoblotting with anti SYT1 and SYT2 antibodies (left). Quantification of SYT1 and SYT2 immunoreactivity was carried out by densitometric scanning and interpolation of the data into a standard curve of rat brain LS1 fraction and expressed in percent of the total input added to the samples (right).

Based on our previous data (Bragina et al., 2007, 2010, 2012) we analyzed SYT1 and 2 in VGLUT1+ and VGLUT2+ terminals; Rab3a and c in VGLUT1+, VGLUT2+, and VGAT+ terminals; and STX1A and B in VGLUT1+ and VGLUT2+ terminals. In all series, we also verified preliminarily the colocalization of each isoform in puncta expressing the different vesicular transporters. The results were in line with published data (Bragina et al., 2010, 2012) (Figures 1A,B).

SYT series showed that ~55% of glutamatergic (VGLUT1+ and VGLUT2+) terminals expressed only isoform 1, ~13% express both isoforms (~75% of isoform 2 appeared coexpressed with isoform 1), and ~25% of glutamatergic terminals express neither SYT1 nor SYT2 (Figure 1B and Table 1). Rab3 isoforms are differentially expressed in VGLUT1, VGLUT2 and VGAT+ terminals: ~60% of VGLUT1+ terminals express only isoform Rab3a, ~8% express both Rab3a and Rab3c (the vast majority of isoform c is coexpressed with isoform a), and ~30% of VGLUT1+ terminals expressed neither Rab3a nor Rab3c (Figure 1B, Table 1). As for VGLUT2+ terminals, ~20% of them express Rab3a, ~12% express both Rab3a and Rab3c (about 50% isoform c colocalizes with isoform a), and ~60% simply lack these Rab3 isoforms (Figure 1B, Table 1). Finally, ~40% of VGAT+ terminals express Rab3a, ~30% express Rab3c, and ~30% express both isoforms (Figure 1B, Table 1). Regarding STX1A and B isoforms, our analysis shows that ~50% of VGLUT1+ terminals express both isoforms, <15% express either isoform, and ~40% express neither STX1A nor STX1B. Most (~65%) VGLUT2+ terminals express neither STX1A nor STX1B, ~15% of them express both STX1A and STX1B, while 15% express only STX1B (Figures 1A,B, Table 1).

**Table 1:** **Quantitation of triple labeling studies**.

	**VGLUT1**		**VGLUT2**		**VGAT**	
SYT1	68.68 ± 7.58	11.99 ± 4.38	71.29 ± 1.53	14.13 ± 1.51	nd	
SYT2	16.16 ± 3.47		18.49 ± 0.49			
Rab3a	66.05 ± 6.00	8.30 ± 4.14	32.80 ± 2.18	12.31 ± 1.35	66.21 ± 6.05	27.67 ± 6.09
Rab3c	11.83 ± 5.03		22.22 ± 5.65		60.03 ± 4.62	
STX1A	59.03 ± 4.99	48.88 ± 5.35	20.93 ± 1.69	15.62 ± 1.56	nd	
STX1B	52.37 ± 4.80		30.28 ± 2.11			

Values (means ± SEM) refer to the percentage of positive puncta for the respective protein isoforms in the three terminal populations.

## Two isoforms of a presynaptic protein on one synaptic vesicle?

Analysis of SYT1 and SYT2 expression indicates that SYT2+ terminals coexpress SYT1. SYT1/SYT2 coexpression may reflect the existence of synaptic vesicles (SVs) expressing both isoforms and that of different pools of SYT+ SVs in axon terminals.

To shed some light on this unexpected finding, we performed immunoisolation studies of rat neocortical (LS1 fraction) SVs expressing SYT1 to establish if the colocalization of SYT1 with with SYT2 occurs on the same vesicles. The enriched fractions (PELLET), together with supernatant fractions (SUP) and total crude vesicular fractions (INPUT) were immunoblotted for SYT1 and SYT2 (Figure 1C). The fraction immunoisolated for SYT1 showed strong labeling for SYT1, whereas the supernatant fraction hardly showed any SYT1 staining, indicating the quantitative isolation of SYT1-containing vesicles (91% of total input). The immunoisolation also resulted in a good co-purification of SYT2 (66% of total input), confirming the substantial coexpression of SYT2 with SYT1 at the synaptic vesicle level. VGLUT1, VGLUT2, and VGAT immunoblotting performed on the same SYT1-immunoisolated samples (data not shown) revealed a similar enrichment of glutamatergic and GABAergic vesicles (62 and 53% for glutamatergic and GABAergic vesicles, respectively).

Synaptotagmins form Ca^2+^-indipendent multimers on SVs surface, resulting in protein complexes in which each subunit binds Ca^2+^ ions (Fernandez-Chacon and Sudhof, 1999). The present demonstration that a large amount of SVs express both SYT1 and SYT2 suggests that SYT oligomers can be composed, to a large extent, by both SYT1 and SYT2.

## Conclusion(s)

The present study was prompted by the need of verifying the assumption that in a given population of axon terminals the sum of terminals expressing different isoforms of a given presynaptic protein (either vesicular or of the plasma membrane) accounts for the whole population of terminals. Recent data gathered in our laboratories allow some initial stimulating conclusions: (1) the possibility that not all families of presynaptic proteins are expressed in all terminals must be taken into serious account. Clearly, we cannot rule out the possibility that other isoforms of a given presynaptic protein (either not tested in the present analysis or still unknown) are expressed at terminals apparently not expressing that protein; (2) conversely, the expression of a given protein isoform in a terminal does not exclude the expression of other isoforms of the same protein in the same terminal. The two cases are well exemplified by the distribution of STX1A and 1B in VGLUT1+ terminals: ~60% of VGLUT1+ terminals express STX1A while ~50% express STX1B, and triple-labeling studies show that ~50% of VGLUT1+ terminals express both isoforms, <15% express isoform A or B, and ~40% express neither STX1A nor STX1B; (3) in addition, the results of the immunoisolation studies, showing that a large percentage of SYT2 is co-expressed in SYT1-immunoisolated vesicles from rat neocortex imply that, within a single synapse, both proteins are sorted to the same synaptic vesicle.

The present observations indicate that molecular heterogeneity of glutamatergic and GABAergic synapses is by far more complex than previously thought. Thus, a combinatorial profile of the nerve terminal complement of protein involved in synaptic transmission can be figured out, with a changing spectrum of physiological properties among different neuronal populations, among neurons belonging to the same population (e.g., interneurons) or even among distinct nerve terminals belonging to the same neuron. The presynaptic protein heterogeneity can affect multiple properties of neurotransmitter release. For example, the distinct distribution of SNAP-25 isoforms, with SNAP-23 replacing the more widespread SNAP-25 in mature inhibitory neurons (Verderio et al., 2004) impacts with the Ca^2+^-dependence of release and the probability of release. Indeed, while SNAP-25 interacts with N− and P/Q-type Ca^2+^ channels and has an inhibitory action on the Ca^2+^ influx in response to depolarization (Wiser et al., 1996; Zhong et al., 1999), SNAP-23 has no such effect, resulting in higher Ca^2+^ influx and higher probability of release in inhibitory neurons than in excitatory neurons (Pozzi et al., 2008). Another interesting functional aspect in which a distinct proteomic spectrum of the nerve terminal can play a major role is the ratio between synchronous and asynchronous release. High frequency stimulation trains lead to delayed asynchronous release in excitatory and inhibitory synapses (Atluri and Regehr, 1998; Lu and Trussell, 2000). While some presynaptic proteins such as synaptotagmin-1, synapsin I and VAMP2 drive synchronous release, their isoforms synaptotagmin-7, synapsin II and VAMP4 are essential for asynchronous release (Geppert et al., 1994; Nishiki and Augustine, 2004; Maximov and Sudhof, 2005; Wen et al., 2010; Raingo et al., 2012; Medrihan et al., accepted). As long-lasting asynchronous GABA release can increase the effectiveness of inhibition (Lu and Trussell, 2000; Manseau et al., 2010), the isoform expression pattern plays a pivotal role in the synchronous-to-asynchronous release ratio and thereby in the control of excitability exerted by subpopulations of inhibitory neurons.

These conclusions open new and interesting problems; among these, the following appear of some interest. Since proteins that are not expressed in certain terminals play a role in synaptic plasticity (Fernandez-Chacon and Sudhof, 1999; Schlüter et al., 2004; Fujiwara et al., 2006), is it possible that different terminals display distinct presynaptic mechanisms participating in short- and long-term plasticity paradigms? Given that it is possible to facilitate or depress synaptic terminals of the same neuron independently of each other (Katz et al., 1993), is it conceivable that neurons synthesize all isoforms redundantly whereas only some terminals express them? Do axon terminals with more than one isoform of a given presynaptic protein express specific combination of isoforms, thus varying their efficiency in the formation of protein core complexes essential for vesicle exocytosis (Pérez-Branguli et al., 1999)? Are SVs with redundant isoforms more likely to contribute to the formation of new synapses (Darcy et al., 2006; Staras et al., 2010; Dobie and Craig, 2011)? How this *scenario* can be modified in diverse physiological conditions, including activity-dependent plasticity? How does the widespread presynaptic heterogeneity modify excitation/inhibition balance, thereby contributing to pathophysiology of neuropsychiatric diseases (Yizhar et al., 2011) and to cognitive decline of brain aging (Pinto et al., 2010; Hickmott and Dinse, 2012)? Future research will shed light on these challenging questions that are fundamental to our understanding of information processing in the brain.

### Conflict of interest statement

The authors declare that the research was conducted in the absence of any commercial or financial relationships that could be construed as a potential conflict of interest.
